# Case report: Atypical neurofibromatous neoplasm with uncertain biological potential of the sciatic nerve and a widespread arteriovenous fistula mimicking a malignant peripheral nerve tumor in a young patient with neurofibromatosis type 1

**DOI:** 10.3389/fonc.2024.1391456

**Published:** 2024-05-10

**Authors:** Nadja Grübel, Gregor Antoniadis, Ralph König, Christian Rainer Wirtz, Juliane Bremer, Andrej Pala, Melanie Reuter, Maria Teresa Pedro

**Affiliations:** ^1^ Peripheral Nerve Unit, Department of Neurosurgery, Bezirkskrankenhaus (BKH) Günzburg at Ulm University, Günzburg, Germany; ^2^ Institute of Neuropathology, University Hospital Rheinisch-Westfälische Technische Hochschule (RWTH) Aachen, Aachen, Germany; ^3^ Department of Neuroradiology, BKH Günzburg at Ulm University, Günzburg, Germany

**Keywords:** atypical neurofibromatous neoplasm with uncertain biological potential (ANNUBP), neurofibromatosis type 1, arteriovenous fistula, neurofibroma, malignancy

## Abstract

We report an unusual constellation of diseases in a 32-year-old woman with neurofibromatosis type 1 (NF1) diagnosed with the recently described precursor entity of malignant peripheral nerve sheath tumor (MPNST), the so-called atypical neurofibromatous neoplasm with unknown biological potential (ANNUBP) and a large symptomatic cervical arteriovenous fistula. An [^18^F] 2-Fluoro-2-deoxy-D-glucose PET/CT (FDG-PET/CT) was performed to detect and stage a conspicuous symptomatic cervical tumor. The FDG-PET/CT showed high FDG uptake in one of the multiple known tumorous lesions associated with peripheral nerves. However, no relevant FDP uptake was observed in this affected cervical area. After digital subtraction angiography, the cervical mass turned out to be a widespread arteriovenous fistula of the vertebral artery. This was successfully treated using endovascular embolization. Subsequently, magnet resonance imaging (MRI) of the FDG-positive tumor revealed a well-enhanced homogeneous mass of the sciatic nerve measuring 5.2×2.4×2.8 cm. Microsurgical gross total tumor resection was performed using ultrasound. The final histopathological diagnosis was ANNUBP transformed from neurofibroma. The patient benefited excellently from the surgery; no recurrence or metastasis has been observed since resection. According to imaging, ANNUBP can be characterized as a well-enhanced homogeneous mass on MRI, displaying high uptake on FDG-PET/CT and hypoechogenic in ultrasound.

## Introduction

Neurofibromatosis type 1 (NF1) is a rare autosomal dominant genetically determined condition. NF1-associated nerve sheath tumors include cutaneous, subcutaneous, intraneural, and plexiform neurofibromas (1, 2). It is common for NF1 patients to develop multiple neurofibromas with a lifetime risk of malignant transformation into malignant peripheral nerve sheath tumors (MPNST) from a preexisting neurofibroma over the course of the illness. This transformation typically occurs in plexiform neurofibromas and is estimated to be between 8% and 15% (2–6). Malignant transformation can occur at any age but is mainly diagnosed between the ages of 30 and 40 (3). With the diagnosis of a high-grade MPNST, the expected 5-year overall survival rate decreases drastically to approximately 20%–50% (7, 8). Therefore, prompt diagnosis followed by appropriate treatment is crucial. Currently, multiple risk factors and diagnostic modalities for the development of malignancy continue to be missing. [^18^F] 2-Fluoro-2-deoxy-D-glucose PET/CT (FDG-PET/CT) is a useful non-invasive imaging modality for discrimination between benign and malignant tumorous lesions in NF1 patients. However, one of the significant limitations is a missing maximum standardized uptake value (SUV_max_) cutoff point (9, 10). In a neuropathology consensus meeting in 2016, the malignant transformation of an “atypical neurofibroma” was categorized into three categories: 1. neurofibroma with cytologic atypia or hypercellularity, 2. atypical neurofibromatous neoplasm with uncertain biological potential (ANNUBP), and 3. MPNST (3). The proposed definition for the term ANNUBP is a neurofibromatous tumor with at least two of the four following features: cytologic atypia, loss of neurofibroma architecture, hypercellularity, mitotic index >1/50 per high power field (HPF), and <3/10 HPF (2, 3). Accurate and precise diagnostics of ANNUBP and differentiation from MPNST are crucial for therapeutic strategies (8). ANNUBPs can be treated with complete resection and are known to be less aggressive with lower risks of metastasis and recurrence than MPNST (3, 11). The diagnosis of ANNUBP is a new and rare entity; only a few case reports present clinical and imaging findings, therapeutic strategies, and clinical outcomes. Despite the referred possibility of malignant transformation of neurofibromas in NF1 patients, there are other challenges in the diagnosis and treatment of NF1 patients, which include other pathologies associated with NF1, for example, skin alterations, gliomas, seizures, scoliosis, learning difficulties, and, in rare cases, vascular malformations such as arteriovenous malformations, cervical artery aneurysms, and arteriovenous fistulas (12, 13).

Herein, we present the disease progression of a young NF 1 patient with an arteriovenous fistula of the vertebral artery mimicking a malignant peripheral nerve tumor and the diagnostic process of an ANNUBP of the sciatic nerve containing the clinical, neurological symptoms, histopathological analysis, imaging modalities including MRI, FDG-PET/CT, and ultrasound and the outcome.

## Case presentation

**Figure 1:**
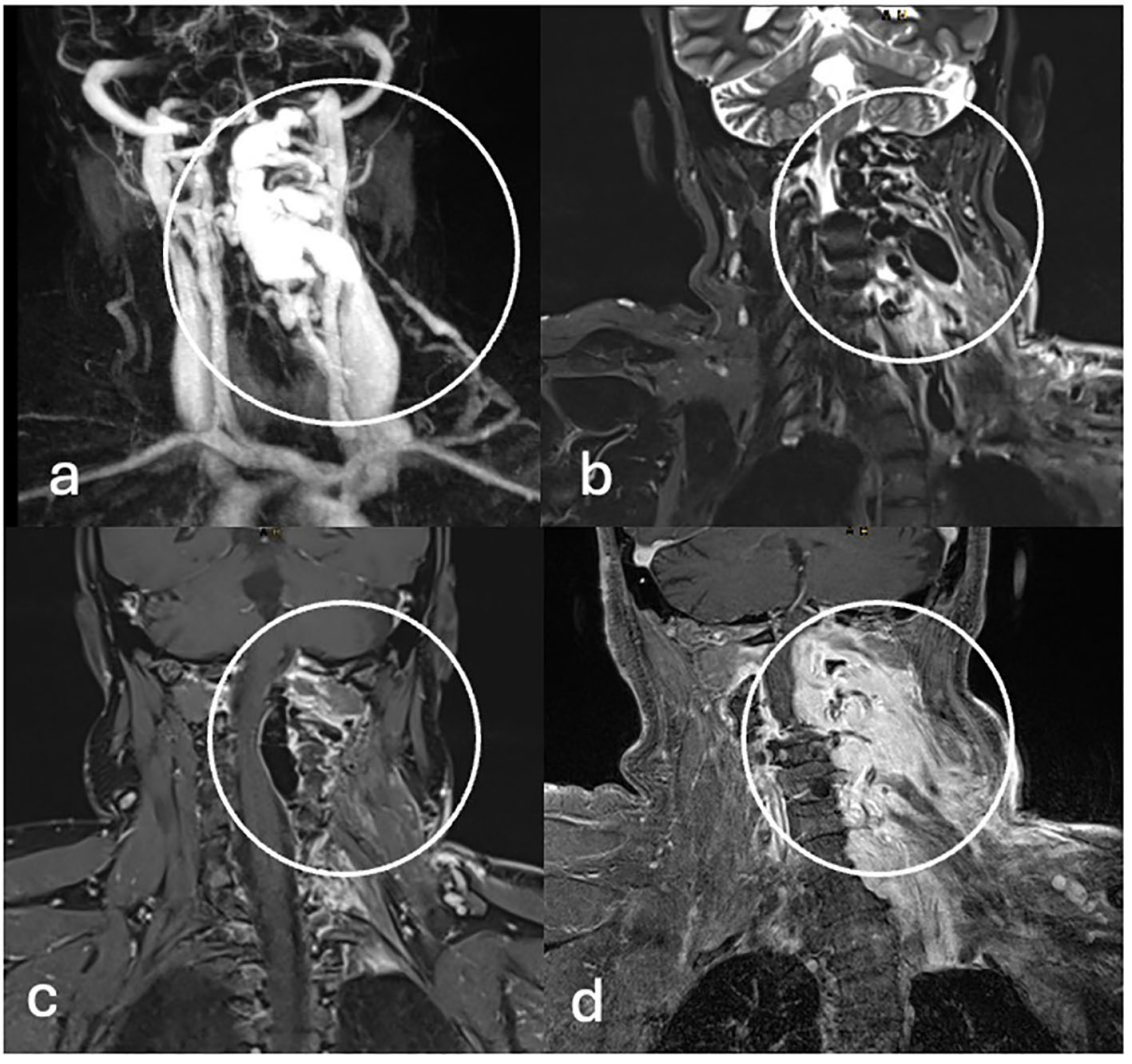
Three-Tesla MRI. **(A)** Coronal twist MR-A, **(B)** T2-weighted, **(C, D)** and T1 weighted MRI with gadolinium enhancement demonstrates an arteriovenous fistula originating of the left vertebral artery with early filling of intra- and extraspinal venous vascular convolutes (circle).

**Figure 2:**
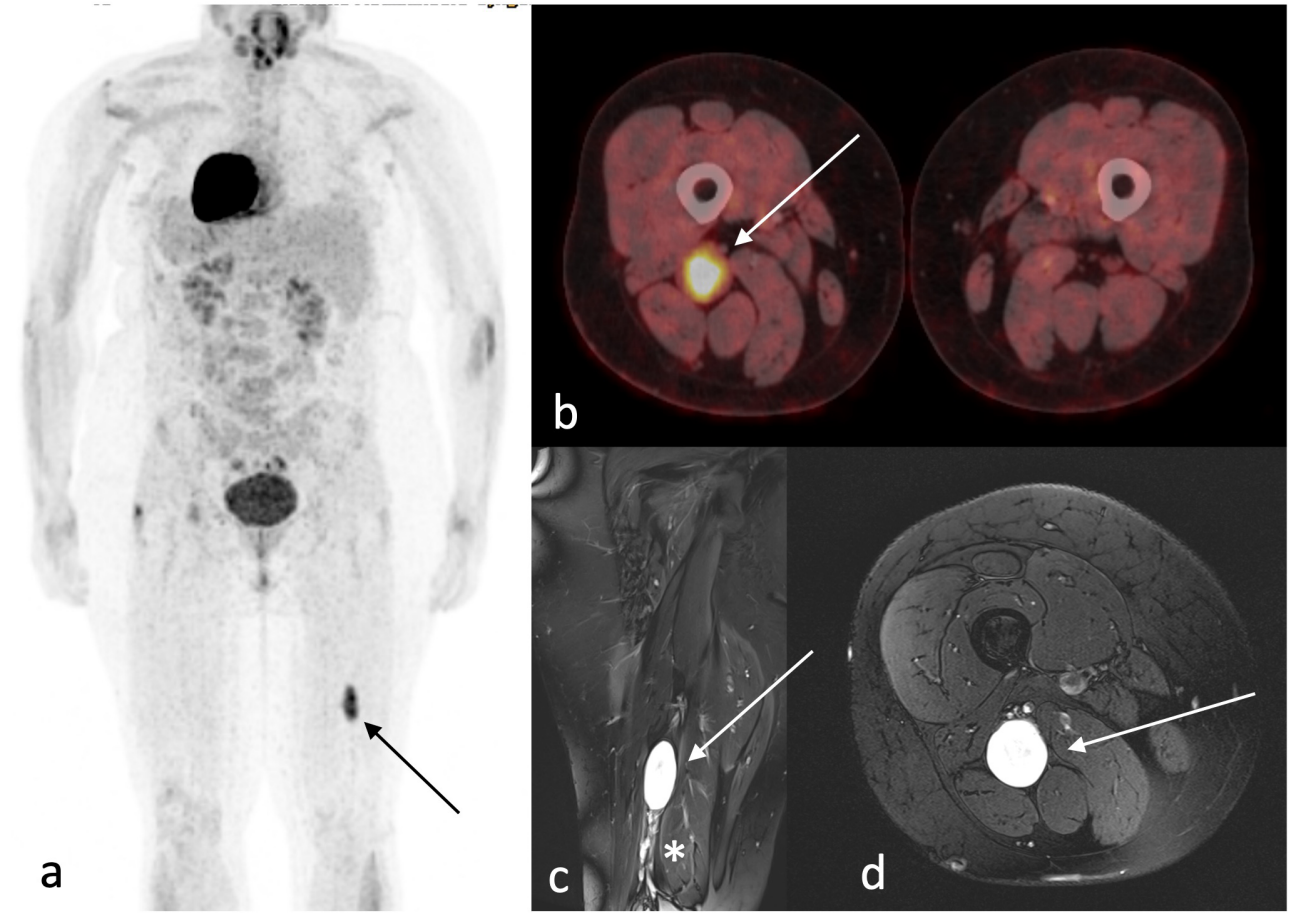
Imaging. ^18^F-FDG-PET/CT **(A, B)** demonstrates FDG accumulation in the right sciatic nerve (arrow) with an SUV_mean/max_ of 6.8/11.7. Coronar **(C)** and axial **(D)** view of T1-weighted gadolinium-enhanced MRI shows a homogenous gadolinium-enhanced 5.2×2.4×2.8 cm large mass arising from the dorsal parts of the sciatic nerve (arrow) in the distal third of the thigh—additional multiple small neurofibromas throughout the sciatic nerve (*), intramuscular and subcutaneous are visualized.

**Figure 3:**
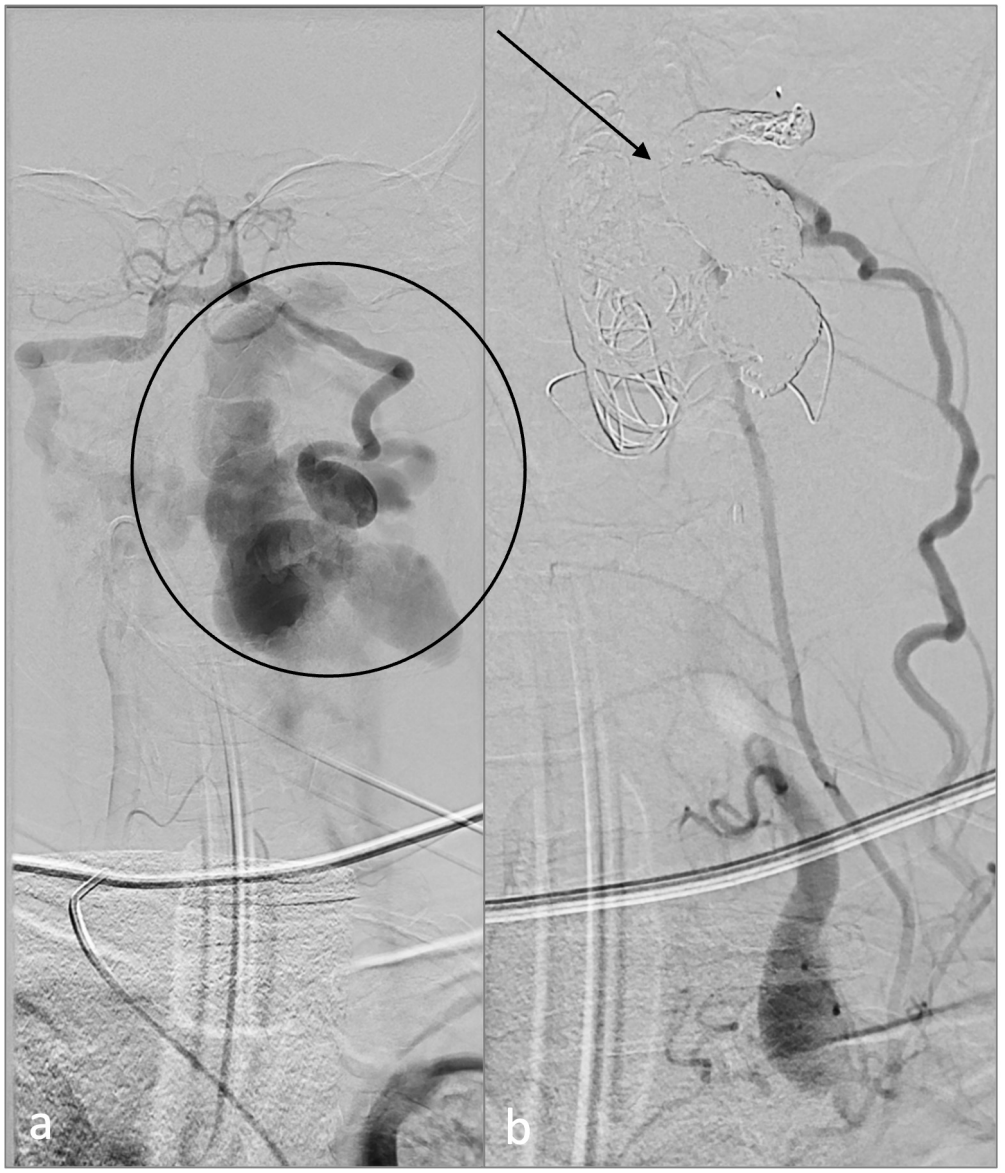
Endovascular treatment. Digital subtraction angiography demonstrates the vertebral arteriovenous fistula **(A)** before (circle) and **(B)** after treatment (arrow).

**Figure 4:**
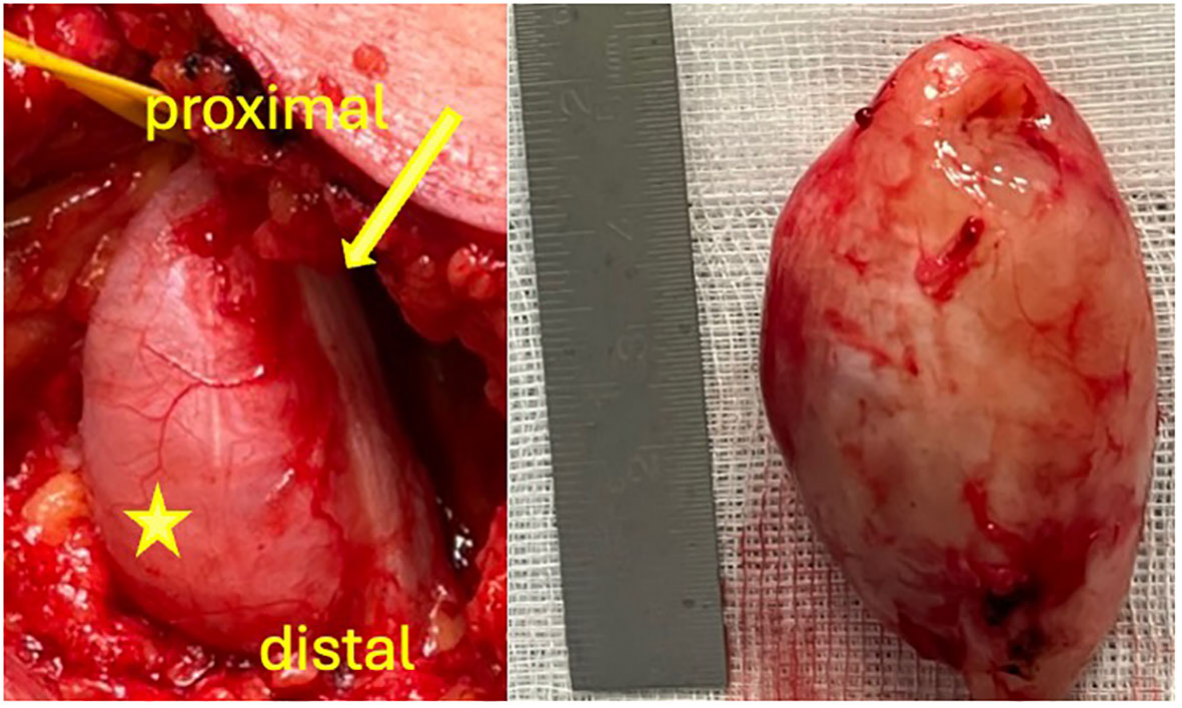
Intraoperative images. Macroscopic appearance of the tumor (asterisk) originating from the sciatic nerve (arrow) *in situ* and after resection.

For fifteen years, a nowadays 32-year-old woman with NF1 has been treated at our department. Initially, in 2008, as the first surgical intervention, a pilocytic astrocytoma was resected, and in 2015, brain tumor recurrence led to a second surgery. She reveals multiple café-au-lait spots on her skin and numerous subcutaneous masses on her whole body as the classical NF stigmata; because of new, immobilizing neck pain and intractable cervicobrachial pain in her left arm, magnet resonance imaging (MRI) of the cervical spine was performed (**Figure 1**), which revealed a long-distance contrast-enhancing mass along the cervical spine. The diagnosis of a malignant peripheral nerve tumor (MPNST) was assumed. Consequently, an FDG-PET/CT was performed to acquire a whole-body staging and evaluate a possible biopsy target (**Figure 2**). Surprisingly, this tumor mass presented no relevant FDG uptake, revealing an SUV_mean/max_ of 2.9/5.3. Instead, a relevant SUV uptake was incidentally found on one of the multiple known peripheral nerve tumors. This tumor rising out of the sciatic nerve on the right side, with a size of 5.2×2.4×2.8 cm, had a high homogeneous-FDG uptake with an SUV_mean/max_ of 6.8/11.7. These results were presented and discussed in a multidisciplinary tumor board, including neuroradiology. Magnet resonance angiography (MR-A) (**Figure 1**) and computer tomography angiography (CT-A) were performed for further diagnostic workup, with results suggesting the cervical mass to be an arteriovenous fistula. Finally, spinal digital subtraction angiography revealed an intra-, extraspinal, retro- and parapharyngeal arteriovenous fistula with an extension from the foramen magnum to C4, with the primary feeder being the vertebral artery on the left side and small feeders of the cervical ascendence artery and the deep cervical artery (**Figure 3**). The venous drainage follows widespread venous vascular convolutes, especially intraspinal. As the patient continued with intractable pain, retro- and anterograde trans-arterial embolization of the fistula was performed. After assessment of sufficient collateralization, the vertebral artery had to be occluded using 26 coils, two Woven EndoBridge (WEB) devices, and one vascular plug to treat the fistula fully. Furthermore, feeders from the cervical ascendence artery were embolized using five coils. The postinterventional MRI showed a fully closed vertebral artery and no early arteriovenous inflow. The initial space-occupying effect of the intraspinal venous convolutes was documented as thrombosed and, therefore, smaller in size. All in all, there was explicit reduction in size intra- and extraspinal followed by decompression of the cervical spinal cord, especially on the levels C1–2. The patient benefited excellently from embolization with totally reduced pain, so she was able to stop her medication only a few days after radiological intervention. But in between the embolization and surgery of the peripheral nerve tumor, the patient developed new rest and stress pain in her right leg but without further neurological deficits. Microsurgical gross total resection was performed using intraoperative ultrasound and nerve stimulation (**Figure 4**). Ultrasound showed a hypoechogenic signal of the tumor with no cystic components. Microsurgical tumor removal was performed using a nerve stimulation probe. After opening of the epineurium, through stimulation, healthy functional fascicles en passant were identified and preserved. The tumor mass itself revealed no motoric response, so by opening its capsule, the complete mass *in toto* was enucleated.

Postoperatively, the patient benefited again from surgery with immediate reduced leg pain. No neurological deficits occurred.

**Figure 5:**
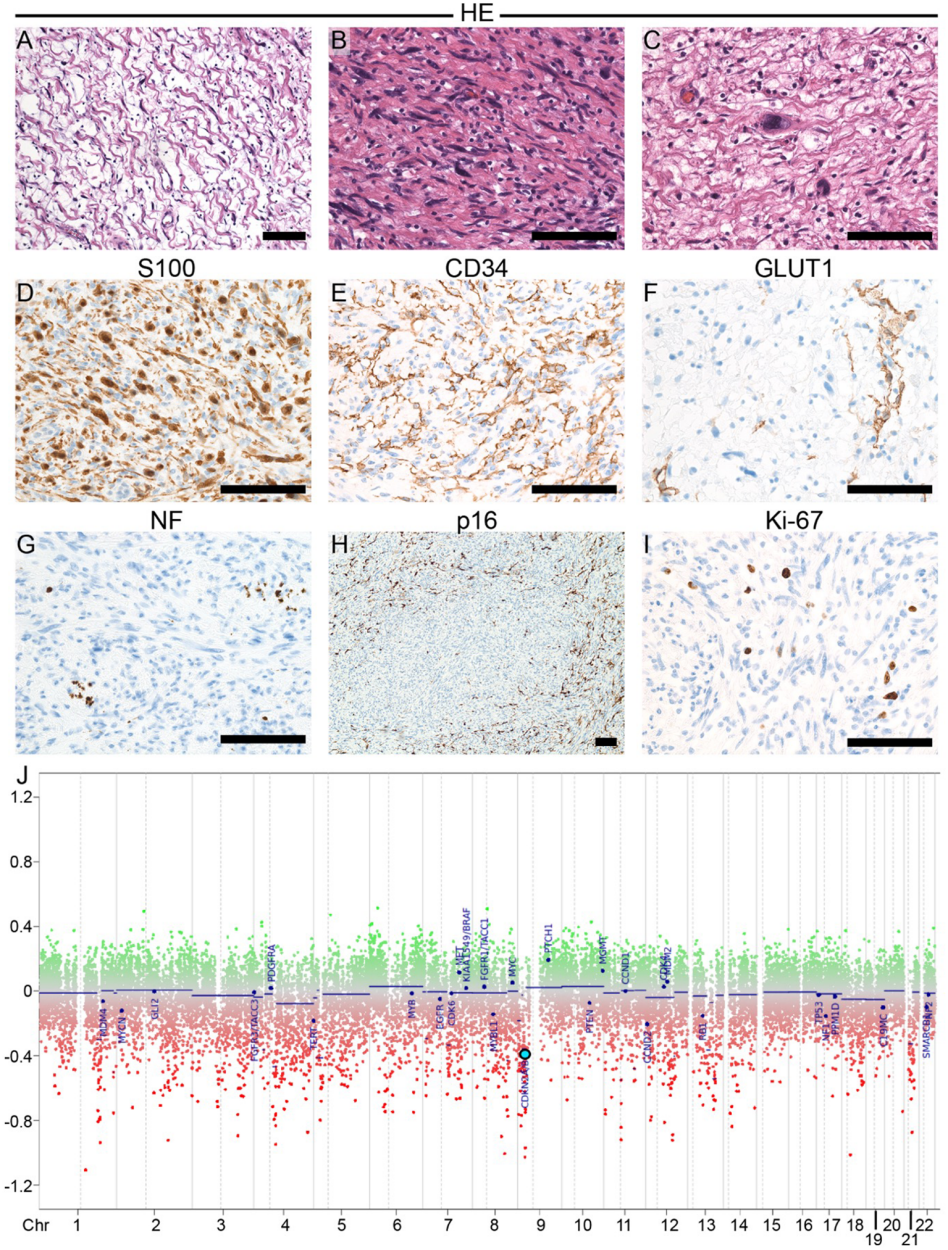
Histopathological analysis and copy number variation (CNV) profile. **(A-I)** Histopathological analysis: H&E stained sections **(A-C)** showing typical histopathological findings of neurofibroma, including cytologically bland, wavy nuclei, loose matrix architecture with collagen bundles, so-called “shredded carrots” in some regions **(A)**; hypercellularity in other regions **(B)** and nuclear atypia **(C)**. Necrosis and increased mitotic activity were absent. Immunohistochemistry **(D-I)** shows that a subset of tumor cells expressed S100 **(D)** and CD34 **(E)**, respectively. A small subset of tumor cells in focal small clusters expressed GLUT1. Entrapped neurofilament (NF) positive axon bundles within the tumor **(G)**. P16, one protein product of the CDKN2A gene, highlights a subset of tumor cells; focal complete loss of p16 was seen **(H)**. Ki-67 proliferation fraction was around 5%. **(J)** The CNV profile of the tumor was generated based on DNA methylation analysis by microarray for 850,000 CpG sites (Infinium MethylationEPIC/ 850k analysis). No chromosomal losses or gains were observed. The CNV profile, however, suggested deletion of CDKN2A/B (light blue dot) on chromosome 9.

Histopathological analysis was initially made at the Department of Neuropathology, BKH Günzburg at Ulm University, Günzburg, Germany. Due to the rarity of the tumor entity and diagnostic uncertainties, referral histology was initiated at the Department of Neuropathology at the University Hospital Aachen, which is particularly specialized in peripheral nerve tumors. Both departments came to the same conclusion: histopathological diagnosis showed a tumor with typical morphological and immunohistological features of neurofibroma in many regions but focally increased cellularity and nuclear atypia. Necrosis and increased mitotic activity, both of which would suggest malignancy, were not seen (**Figure 5**). DNA methylation analysis by microarray (Infinium MethylationEPIC/850k analysis) assigned this tumor to the methylation class “schwannoma” matching score >0.99 (v12.8) and suggested CDKN2A/B deletion (**Figure 5**). Based on these findings and in line with current diagnostic criteria (3, 14), we diagnosed an atypical neurofibromatous neoplasm with uncertain biological potential (ANNUBP). ANNUBP’s often cluster with schwannomas in methylation analysis and harbor CDKN2A/B deletions (14, 15). Adjuvant aggressive treatment could be avoided by early diagnosis and complete resection. To date, 4 months postoperative, no recurrence has been observed. Follow-up digital subtraction angiography is planned 6 months after embolization, an MRI of her right tight is scheduled every 3 months, and FDG PET/CT 12 months after tumor resection.

## Discussion

We report a case of the disease progression of a young female patient with NF1, including the clinical and imaging characteristics of ANNUBP with a high potential for further malignant transformation. The malignant progression of neurofibroma to MPNST in NF1 patients is associated with a worse prognosis (11, 16). Early and accurate diagnosis is crucial yet challenging for those patients with NF1. Up-to-date imaging modalities such as FGD PET/CT are the most valuable tools to detect possible malignant progression (9, 17, 18). Tovmassian et al. found a significant difference between the mean SUV_max_ of benign (SUV_max_, 1.93) and malignant (SUV_max_, 7.48) lesions. A clear SUV_max_ cutoff has yet to be identified (3.1–6.1) (9). Without FDG PET/CT, the relevant peripheral nerve tumor (PNT) would not have been detected in our case. MRI is widely used and reported to help detect malignancy and differentiate benign from malignant neurofibromas (8, 19). The size of the tumor, enhancement pattern, perilesional edema, and intratumoral cystic changes significantly differed between benign and malignant lesions (6, 19). In addition, the apparent diffusion coefficient (ADC) value based on diffusion-weighted imaging (DWI) improved the diagnostic accuracy (19). In our case, preoperative MRI findings show multiple homogenous contrast-enhancing masses on the right sciatic nerve, of which the largest (5.2×2.4×2.8 cm) had high FDG-uptake in the FDG PET/CT. No cystic lesions or perilesional edema were found.

A possible strategy for progress monitoring could be to perform an ultrasound on the most significant, most suspicious nerve tumors in NF1 patients and, in case of enlargement, to perform an FDG PET/CT and evaluate the SUV_max_ uptake. We performed a microsurgical gross total resection using neurostimulation and ultrasound. We could spare functional nerve fascicles, ensuring the preservation of the entire sciatic nerve. Intraoperative, no clues for malignancy were found. In this present case, complete resection of the tumor was preferable to a needle biopsy. In a solitary biopsy, there is a risk of sampling areas that have not yet become malignant, thus leading to an incorrect diagnosis. During the operation, there is no way to distinguish between malignant and potentially non-malignant parts. If the tumor is only partially FDG positive, it cannot be detected intraoperatively. During an ultrasound-guided biopsy, there is a risk of injuring healthy single functional fascicles, leading to subsequent pain or neurological deficits. Only through an open surgical procedure is nerve stimulation feasible, and affected displaced fascicles can be preserved via microsurgical preparation. That is why we prefer generous indications for gross total and early resection of suspicious masses in NF1 patients. By detecting an early malignancy transformation, patients’ overall survival can be longer. In our experience, clinical symptoms such as increasing rest pain, motor weaknesses, and the growth of palpable or visible masses can be a nonnegligible indicator for malignant transformation. Therefore, it is essential to have a short follow-up examination period for patients exhibiting these symptoms. Given the rarity of these lesions and the need to detect clinical changes early, it is reasonable to treat NF1 patients at specialized centers with an interdisciplinary approach.

In our case, clinical deterioration, including rest and stress pain, were indicators of malignant transformation. It can be discussed whether the development of the vertebral arteriovenous fistula should be considered an indicator of clinical deterioration or malignant transformation. Zhao et al. discuss whether vertebral AVF in NF1 patients is innate or if the NF1 disease progresses (13). Our case indicates disease progression. However, due to its rarity, the exact resolution of this debate remains elusive.

## Conclusion

This case emphasizes the complex nature of NF1 and its diagnostic and therapeutic challenges. The use of multiple diagnostic modalities, such as MRI, ultrasound, and FDG-PET/CT, proves to be valuable tools in detecting atypical and malignant transformation of neurofibromas in patients with NF 1. Early histopathological confirmation is vital for overall survival in those patients. Gross total resection at a high volume and specialized center with an interdisciplinary approach might be preferable to a biopsy due to concerns of potential neurological complications.

## Data availability statement

The original contributions presented in the study are included in the article/supplementary material. Further inquiries can be directed to the corresponding author.

## Ethics statement

The studies involving humans were approved by Ethics committee University of Ulm. The studies were conducted in accordance with the local legislation and institutional requirements. The participants provided their written informed consent to participate in this study. Written informed consent was obtained from the individual(s) for the publication of any potentially identifiable images or data included in this article.

## Author contributions

NG: Conceptualization, Data curation, Formal analysis, Investigation, Methodology, Visualization, Writing – original draft. GA: Supervision, Writing – review & editing. RK: Supervision, Writing – review & editing, Data curation, Methodology. CW: Supervision, Writing – review & editing. JB: Conceptualization, Data curation, Formal analysis, Methodology, Visualization, Writing – original draft. AP: Conceptualization, Data curation, Formal analysis, Methodology, Writing – review & editing. MR: Writing – review & editing, Visualization. MP: Conceptualization, Methodology, Validation, Writing – original draft.
